# Excessive Activation of TLR4/NF-κB Interactively Suppresses the Canonical Wnt/β-catenin Pathway and Induces SANFH in SD Rats

**DOI:** 10.1038/s41598-017-12196-8

**Published:** 2017-09-20

**Authors:** Junpeng Pei, Lihong Fan, Kai Nan, Jia Li, Zhibin Shi, Xiaoqian Dang, Kunzheng Wang

**Affiliations:** 1 Department of Orthopaedics, the Second Affiliated Hospital of Xi’an Jiaotong University, No. 157 Xiwu Road, Xi’an710004, Shaanxi Province, People’s Republic of China; 2 Department of Orthopaedics, First Affiliated Hospital of Xi’an Jiaotong University, School of Medicine, No. 277 Yanta Road, Xian, 710061 China

## Abstract

Nuclear factor-kappa B (NF-κB) interactively affects the Wnt/β-catenin pathway and is closely related to different diseases. However, such crosstalk effect in steroid-associated necrosis of femoral head (SANFH) has not been fully explored and evaluated. In this study, early-stage SANFH was induced by two doses of lipopolysaccharide (LPS, 2 mg/kg/day) and three doses of methylprednisolone (MPS, 40 mg/kg/day). LPS and pyrrolidine dithiocarbamate (PDTC) were administered to activate the TLR4/NF-κB pathway and selectively block the activation of NF-κB, respectively. Results showed that PDTC treatment significantly reduced NF-κB expression, diminished inflammation, and effectively decreased bone resorption processes (osteoclastogenesis, adipogenesis, and apoptosis), which were evidently reinforced after osteonecrosis induction. Moreover, PDTC remarkably increased the interfered Wnt/β-catenin pathway and elevated bone formation processes (osteogenesis and angiogenesis). Ultimately, PDTC treatment effectively reduced the incidence of SANFH. Therefore, the excessive activation of TLR4/NF-κB may interactively suppress the Wnt/β-catenin pathway and induce SANFH. Hence, we propose NF-κB-targeted treatment as a novel therapeutic strategy for SANFH.

## Introduction

Corticosteroids have been widely utilized in clinical practice due to their effectiveness in the treatment of manyautoimmune diseases^1^; however, their complications are poorly understood. Given the absence of definite underlying pathogenesis and efficacious therapeutics, steroid-induced avascular necrosis of femoral head (SANFH), a common bony complication of corticosteroid treatment, is causing many patients to suffer from considerable pain and economic losses^2–4^. Recent studies have proven that the pathogenic mechanisms of SANFH are closely correlated with excessive inflammatory immune response^5,6^.

Inflammatory response participates in many pathological processes, respiratory failure, liver dysfunction, and coagulopathy^7^. As the most representative factor of inflammation, nuclear factor-kappa B (NF-κB), a central mediator of inflammation response, determines whether inflammation resolves or progresses to cascaded injuries^8^; NF-κB was hypothesized to be tightly linked to many clinical orthopedic problems, such as osteoporosis, through cytokine release^9,10^. Constitutive activation of NF-κB is related to the degradation of vitamin D-or retinoid X-dependent osteocalcin gene transcription^11^ and plays a pivotal role in the impairment of osteogenesis and skeletal development^12^. The activation of the TLR4/NF-κB pathway is closely associated with rat femur necrosis, and the utilization of its target activator lipopolysaccharide (LPS) can notably increase the incidence rate of SANFH^13^.

Wnt pathways are pivotal in regulating cell proliferation, apoptosis, differentiation, and metabolism, which are responsible for many important and different processes in homeostasis and disease throughout the life cycle of all animals^14^. Aberrant Wnt signaling underlies a wide range of pathologies both in humans and animals, and its various components contribute to the development of different diseases^15,16^. The canonical Wnt/β-catenin pathway, whose transduction cascades control myriad biological phenomena, regulates many bone biology processes, including bone development and remodeling^17^. Inhibiting β-catenin and Wnt signal-related molecular activities in osteoblasts, angiogenesis, and adipogenesis evidently affects bone mass^18,19^. Although the activation of Wnt/β-catenin signaling mediates both osteoblasts and osteoclasts, tendentiously targeting osteocytes by activating the Notch pathwayultimately results in bone gain^20^. In orthopedics, SANFH and osteoporosis are strongly linked to distinctly suppressed β-cateninexpression^21–24^.

TheTLR4/NF-κB and Wnt/β-catenin signaling pathways interactively regulate each other through their pathway processes and independent subset target genes’ functions^25–28^. Depending on these contexts, both positive and negative cross-regulating effects have been observed in cellular or tissue research. Complex crosstalk effects characterized from tissues to organs affect the genesis and development of various clinical diseases, such as cancer, inflammatory, and immune disease. Nevertheless, such crosstalk relationship has not been assessed in SANFH. In this regard, we administered pyrrolidine dithiocarbamate (PDTC) to selectively block the activation of NF-κB during a period to evaluate the underlying crosstalk mechanism of the two pathways. We mainly assessed whether PDTC exerts effect on 1) preventing the genesis and development of SANFH in an early stage; 2) regulating the Wnt/β-catenin signaling pathway through inhibiting the TLR4/NF-κB pathway; and 3) regulating osteogenesis, angiogenesis, osteoclastogenesis, adipogenesis, and apoptosis.

## Results

### PDTC effectively prevented osteonecrotic changes by osteonecrosis induction

**Figure 1:**
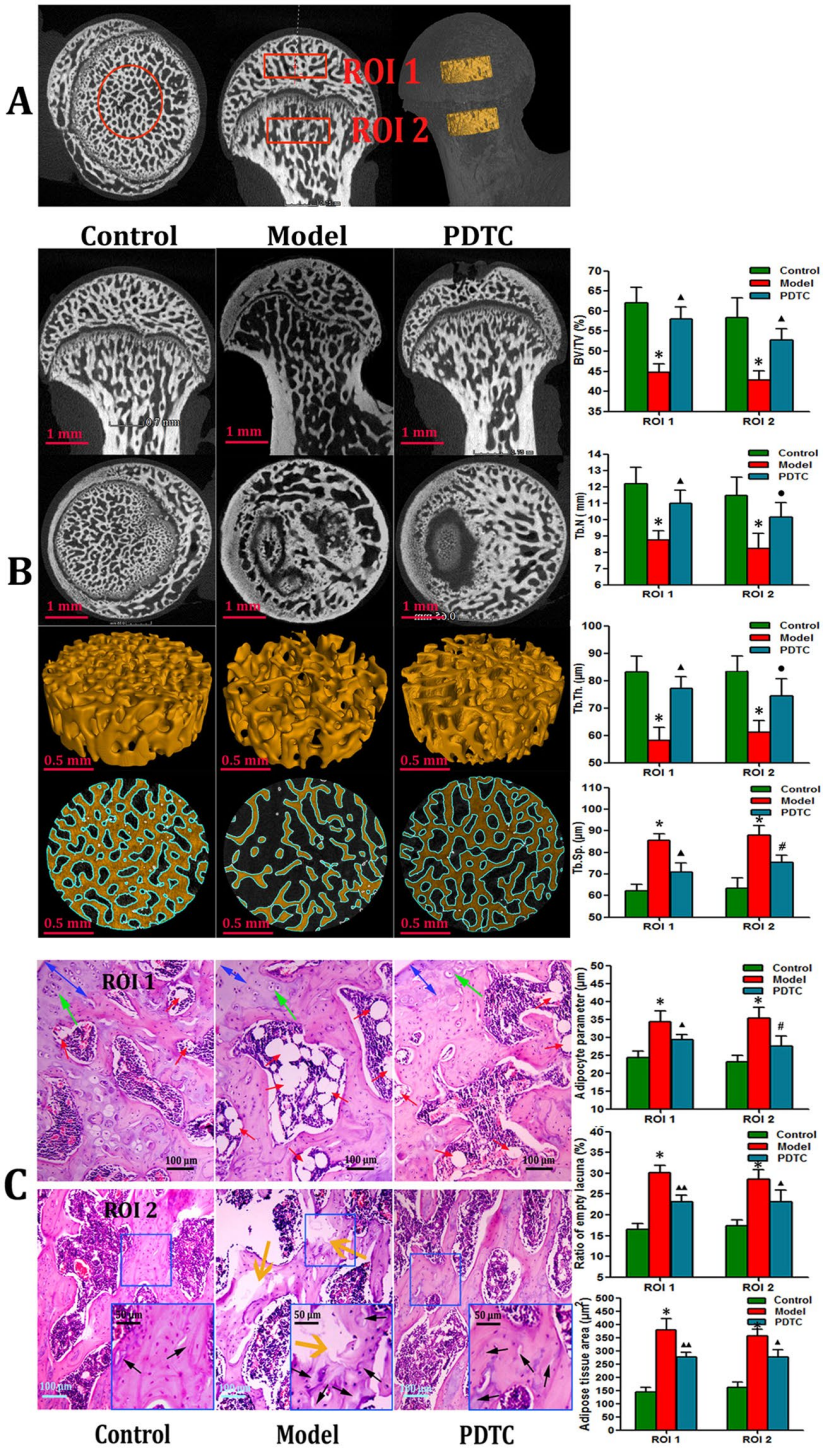
Results of osteonecrotic changes from μ-CT and histological features. (**A**) The selection of regions of interests (ROI 1 and ROI 2). Three-dimensional micron-computed tomography (3D-μCT) ROIs on both sides of gristle of rat femoral heads were selected in the most center of each compartment (calotte and neck). Size selection of cylinder parameters (r = 0.65mm, h = 0.30mm) met fully accommodate to the greatest extent completely. (**B**) Representative μ-CT scanning images and three-dimensional reconstruction results. Compared with the control group, the trabecular bone in the model group was irregular and relatively thin. However, PDTC treatment group showed a better 3D structural integrity of the trabecular bone than that of model group. Bar graphs in the right column shown microstructural parameters of ROI 1 and ROI 2 in different groups, respectively. (BV/TV, bone volume/total volume of bone, expressed as a percent(%); Tb.N, trabecular number, expressed as 1/μm; Tb.Th, trabecular thickness, expressed as μm; Tb.Sp, trabecular separation, expressed as μm. Data illustrated are from three separate samples per group, and represented as the mean ± SD. n **= **3.Tested by Dunnett-t). (**C**) Representative histological features results of osteonecrotic changes from three groups. Samples harvested 6 weeks after osteonecrosis induction. Images gained from HE staining. Line **C1** from calotte (ROI 1) and line **C2** from neck (ROI 2). Histological features of normal bone in control group, osteonecrotic bone model group and the PDTC treatment group, respectively. The normal control group showed a spherically-shaped femoral head, articular cartilage thickness and intratrabecular bone with osteocytes and subchondral bone cells in the marrow were demonstrated (Fig. 1C, blue arrows indicate the thickness of the cartilage, green show single or clustered subchondral bone cells). The presence of neck microfractures were clearly observed in model group (yellow arrows). Statistical analysis on the differences of the ratio of empty lacuna, the adipose tissue area and the adipocyte perimeters as right bar graphs shown. (Original magnification × 100. Quantitative analysis was based on at least 6 fields sections per group. n = 6. Tested by Dunnett-t) (*p < 0.01 v.s. the control; ^▲^P < 0.05 vs. model & control; ^▲▲^p < 0.01 vs. model & control; ^**#**^p < 0.05 vs. model, P < 0.01 vs. control; ^•^p < 0.05 vs. model, P > 0.05 vs. control).

The incidence rates of osteonecrosis were 51.9% (14/27) and 19.2% (5/26) in the model and PDTC groups, respectively. No osteonecrosis was observed in the control group (0/18). Results from μ-CT of selected ROIs (Fig. 1A) showed that the PDTC group exhibited better structural integrity, superior microstructural parameters (BV/TV, Tb.N, Tb.Th, and Tb.Sp), and more regular and thicker trabecular bone than the model group. However, these properties were still relatively inferior to those of control group (Fig. 1B).The histopathological necrosis and repair evolution of the rats were assessed by referring to previous reports^29,30^.

As shown in Fig. 1C, accumulating hypertrophy fat cells (red arrows), thinner cartilage cap (blue double-headed arrow) and microfractures, and debris of bone marrow cells (yellow arrows) were observed in the SANFH lesions of the model group compared with the control group. In addition, the adipose tissue area, adipocyte parameters, and empty lacuna ratio in the bone trabeculae remarkably increased after SANFH induction. Meanwhile, PDTC administration largely attenuated or decreased these increases.

### PDTC ameliorated inflammation response through inhibiting the activation of NF-κB

**Figure 2:**
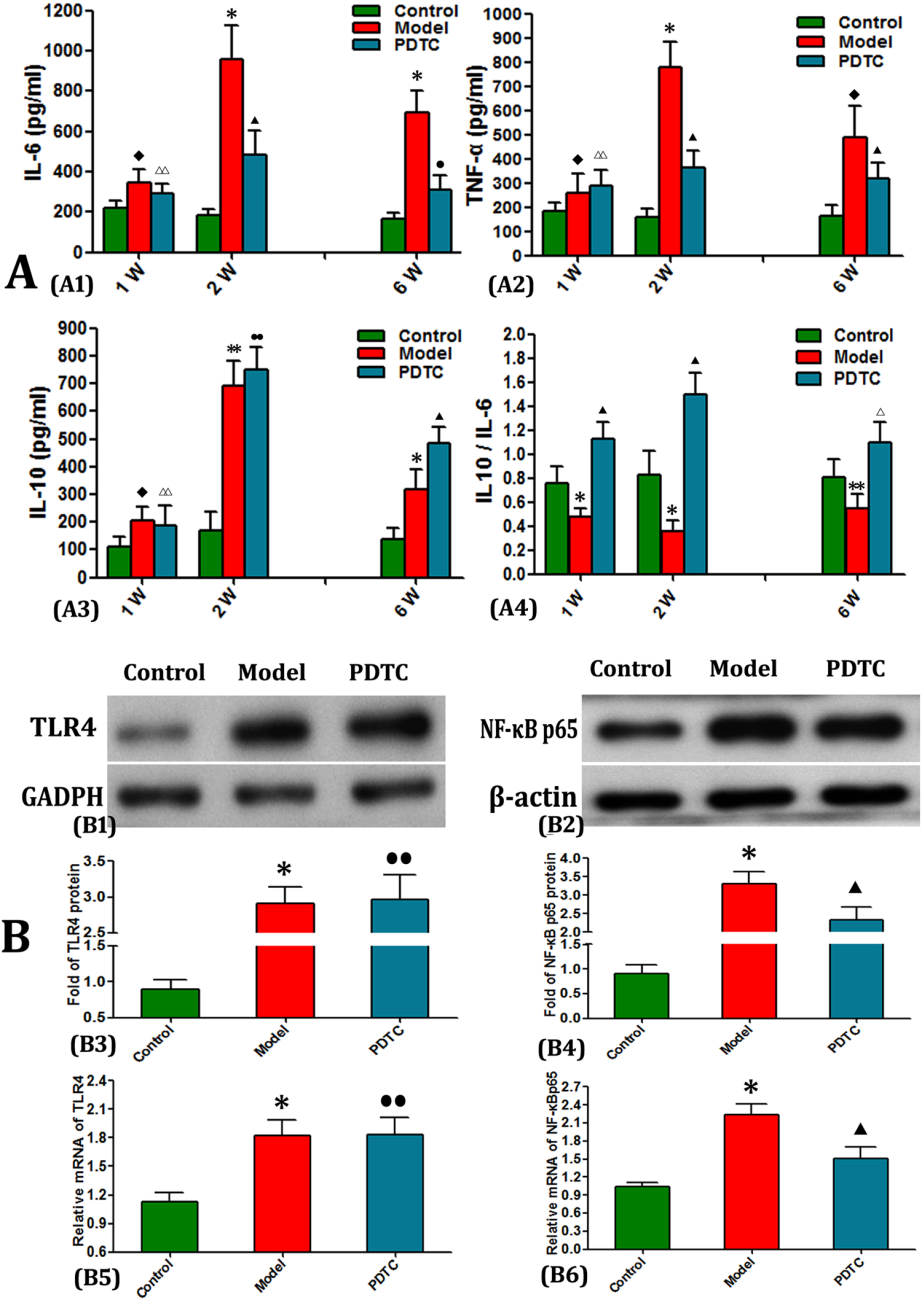
Serum cytokines and TLR4 and NF-κB p65 protein levels in different groups. (**A**) Serum anti- and- pro-inflammatory cytokines of IL- 6 (**A1**), IL-10 (**A3**) and TNF-α (**A2**) were dynamically measured via ELISA assays at 1, 2 and 6 weeks (n = 6). Except the first week, PDTC treatment constitutive suppressed both anti- and- pro-inflammatory cytokines secretion in SANFH. Moreover, the ratio of IL-6/IL-10 (**A4**) was effectively reduced at almost every time points. (n = 6 rats serum per group. Tested by Dunnett-t). (**B**) Expression levels of TLR4 and NF-κB p65. Compared with the control group, high expressions of TLR4 and NF-KB p65 in the femoral heads of SANFH SD rats were detected by Western blot and RT-qPCR. Differently, after PDTC treatment, NF-KB p65 was effectively decreased both at protein and nucleotide expression levels, but no differential found of TLR4 expression. (Data illustrated are from three separate experiments performed on different experimental days. Tested by Dunnett-t.) (*p < 0.01 v.s. the control; **p < 0.05 v.s. the control; ^◆^p > 0.05 v.s. the control; ^▲^P < 0.05 vs. model & control; ^**#**^p < 0.05 vs. model, P < 0.01 vs. control; ^•^p < 0.05 vs. model, P > 0.05 vs. control; ^••^p > 0.05 vs. model, P < 0.01 vs. control; ^**△**^P < 0.05 vs. model, p > 0.05 vs. control).

To explore the relationship of dynamic inflammation change intensity to SANFH, the serum cytokine levels of IL-6, IL-10, and TNF-α were detected at 1, 2, and 6 weeks. The blood chemistry data in the model group internal system revealed that the expression levels of both anti-and proinflammatory factors (IL-6, IL-10, and TNF-α) significantly increased after osteonecrosis induction, especially at 2 and 6 weeks. By contrast, PDTC treatment dramatically reversed these changes. Meanwhile, IL-6/IL-10 ratios evidently decreased almost at all time points (Fig. 2A), suggesting that PDTC exhibits significant anti- inflammatory effects. NF-κB p65 expression was significantly induced in the model group but obviously decreased after PDTC treatment. Although TLR4 was up regulated at the transcription and translation levels in the model group, no significant change was observed in the PDTC group (Fig. 2B).

### PDTC elevated the degradation of Wnt/β-catenin pathway

**Figure 3:**
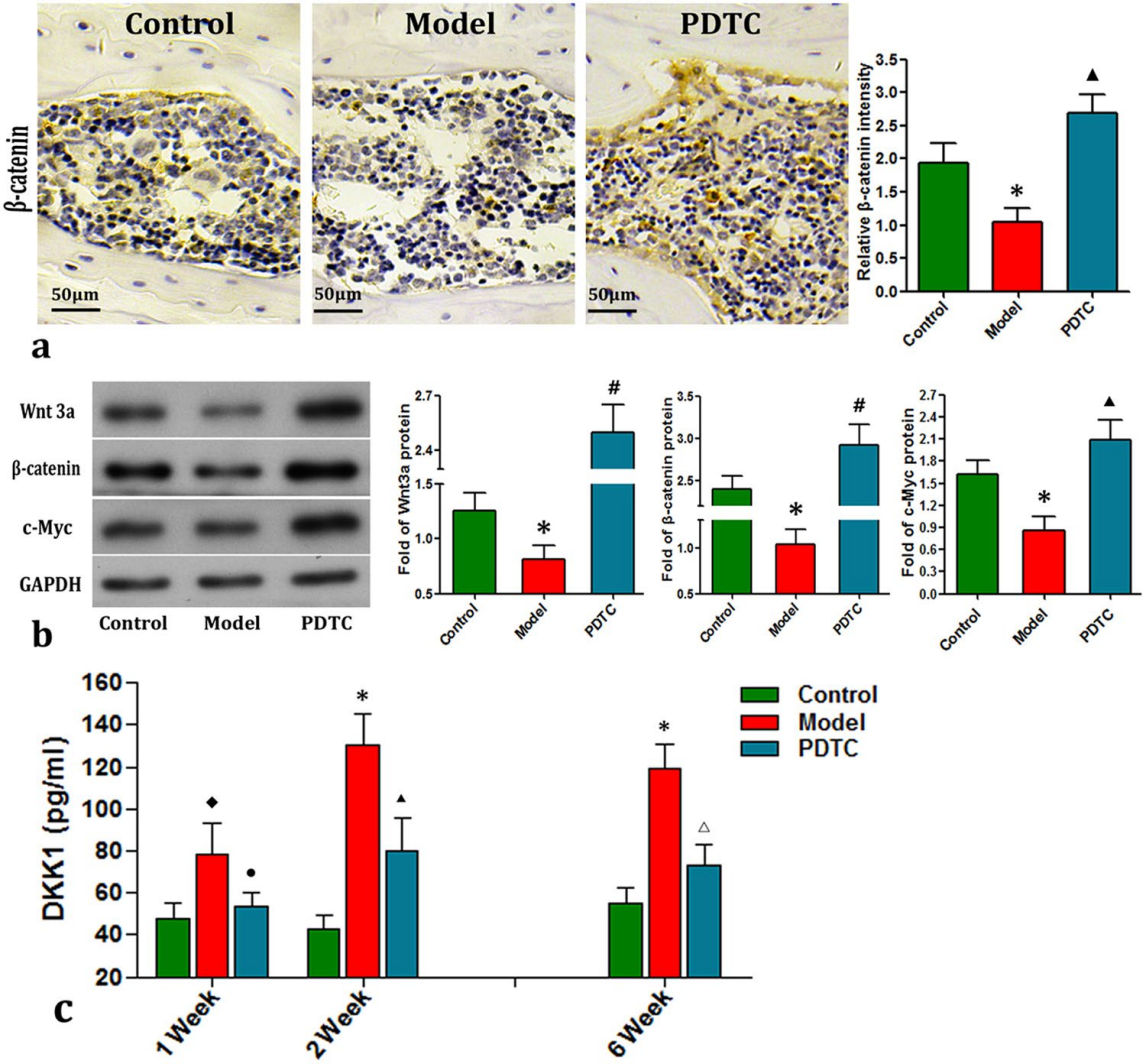
Expressions of Wnt3a, β-catenin, and serum expression level of DKK1. (**a**) Immunohistochemical staining of β-catenin. In the model group, weak immunoreactivity of β-catenin was observed, however, relatively high immunoreactivity intensity of β-catenin was observed after the administration of PDTC. β-catenin was observed in the cell cytoplasm of cartilage, trabecular bone and bone marrow. Bar graphs represent the mean optical density of β-catenin in bone marrow. (Quantitative analysis was based on at least 10 fields per rat. n = 10. Tested by Dunnett-t.). (**b**) Expression levels of Wnt3a, β-catenin and c-Myc by Western blot. The activation of Wnt3a/β-catenin signaling pathway was further confirmed by Western blot analysis. Compared with the control group, the protein expressions of Wnt3a, β-catenin as well as endonuclear reporter c-Myc were significantly decreased. While, PDTC treatment congruously elevated their expressions, even higher than these of control group. (Data illustrated are from three separate experiments performed on different experimental days. Tested by Dunnett-t.) (**c**) The serum DKK1 by ELISA. The serum expression level of Wnt antagonist DKK1 was detected by ELISA. Conversely with Wnts, serum DKK1 constitutively increased after osteonecrosis induction, and effectively desminised after PDTC treatment. (n = 6 rats serum per group. Tested by Dunnett-t.) (*p < 0.01 v.s. the control; ^◆^p > 0.05 v.s. the control; ^▲^P < 0.05 vs. model & control; ^▲▲^p < 0.01 vs. model & control; ^**#**^p < 0.05 vs. model, P < 0.01 vs. control; ^•^p < 0.05 vs. model, P > 0.05 vs. control; ^**△**^P < 0.05 vs. model, p > 0.05 vs. control.).

The results of immunohistochemical staining, Western blot analysis, and RT-qPCR analysis (Fig. 3) congruously showed that 14 doses of PDTC effectively elevated the expression levels of Wnt3a, β-catenin, and c-Myc in the PDTC group compared with the model group. Also, higher than that of control group, showing an enhancement effect of Wnt/β-catenin pathway in pathological condition. Adversely, the serum level of Dickkopf (DKK1) protein substantially increased in the model group during and after acute inflammation. PDTC effectively prevented such increase (P < 0.05), which indirectly reflects the constitutive suppression of the Wnt/β-catenin pathway.

### PDTC treatment enhanced osteogenesis and promoted angiogenesis

**Figure 4:**
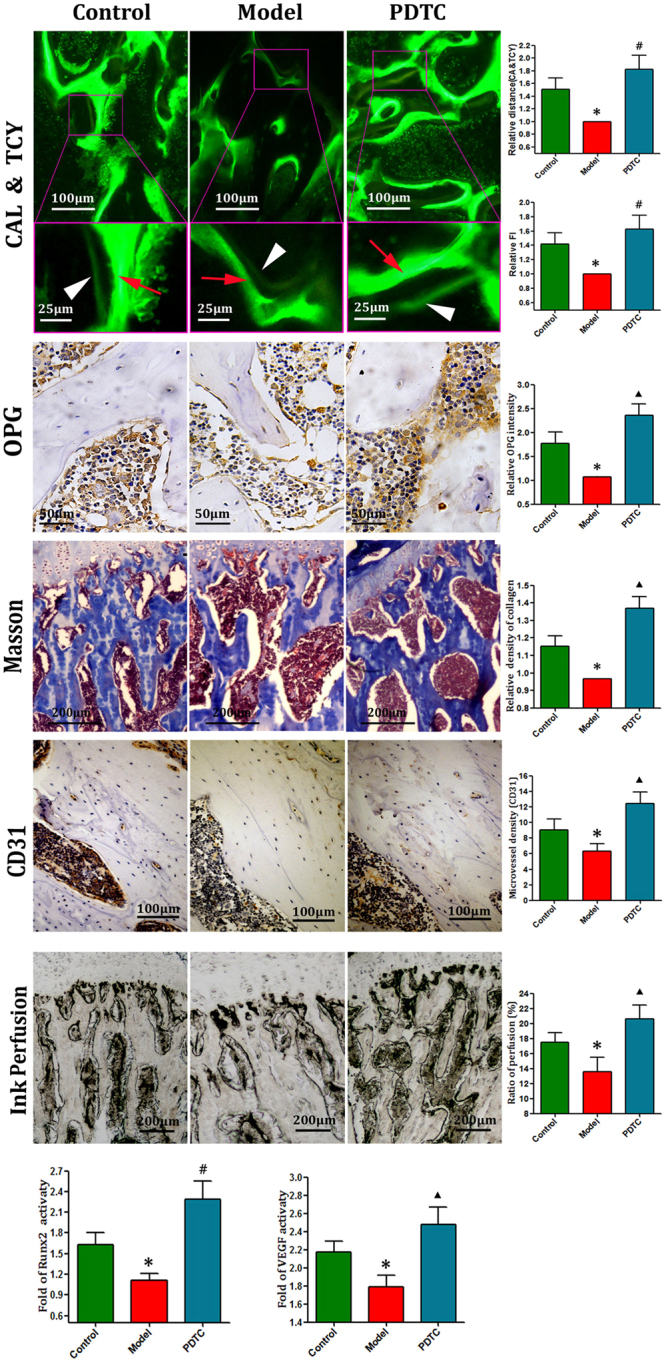
Osteogenesis & angiogenesis. Line 1. Calcein-Tetracycline double labeling of new bone formation. Representative images of the central femurs from SD rats by fluorescence microscopy. The distance between CAL and TCY strips (yellow inside and green outside, respectively) of PDTC group were much wider than model group, and generally close or wider with that of control group. Bars show that the fluorescence intensity comparisons demonstrated a similar result. Line 2&3. Immunohistochemical staining of OPG, and Masson staining. The administration of LPS + MPS significantly suppressed the expressions of OPG and collagen in the model group. However, PDTC treatment effectively reversed the degradation, even superior to the control group. Bar graphs represent the mean optical density of OPG, collagen in bone marrow. Line 4&5. Immunohistochemical staining of CD31 and Ink artery infusion of the femoral head. Images of immunohistochemical staining of CD 31 and ink artery infusion. Few blood vessels and less ink perfusion area were found in the model group, however, more stained blood vessels and much more perfusion ratio were detected in the PDTC group. These results even superior in the PDTC group to control group. Bar graphs show the numeber of blood vessels and ratio of perfusion in the femoral head. Line 6.  Expression levels of Runx2 and VEGF by RT-qPCR. The mRNA expression levels of Runx2 and VEGF activations were further confirmed by RT-qPCR. Compared with the control group, the expressions of Runx2 and VEGF were significantly decreased in the model group. While, PDTC treatment efficiently elevated the expressions of VEGF and Runx2. (Above staining quantitative analysis parameters were based on at least 10 fields <n = 10**>**. Data from RT-qPCR illustrated are from three separate experiments. Bars show relative values of three groups. *p < 0.01 v.s. the control; ^▲^P < 0.05 vs. model & control; ^**#**^p < 0.05 vs. model, P < 0.01 vs. control. Further tested by Dunnett-t).

The abilities of bone remodeling were detected by performing CAL-TCY double labeling, immunohistochemical staining of OPG and CD31 proteins, Masson staining of collagen, ink artery infusion of femoral artery, and RT-qPCR analysis of Runx2 and VEGF expression. Figure 4 shows that the distance between CAL and TCY strips and the fluorescence intensity (FI), the expression levels of OPG and CD31 positive cell numbers, and the ink perfusion ratio of artery sharply decreased after SANFH induction. However, PDTC treatment reversed such down regulation effectively; considerably wide distance between CAL and TCY strips, high FI, many OPG-positive cell numbers, and many mature capillaries and single or cluster endothelial cells (CD31) separated from adjacent microvessels were clearly observed in the PDTC group. The artery ink infusion ratio also increased remarkably when compared with the model group (all P < 0.05). Masson staining of fibrotic tissue showed a striated blue. Similar results showed that deep dyeing collagen was observed in the PDTC group. The most sparse collagen staining demonstrated the osteogenesis inability of the model group. These positive regulation effects were verified reliably and congruously at the nucleotide expression levels of Runx2 and VEGF by RT-qPCR.

### PDTC suppressed osteoclast activity and attenuated adipogenesis

**Figure 5:**
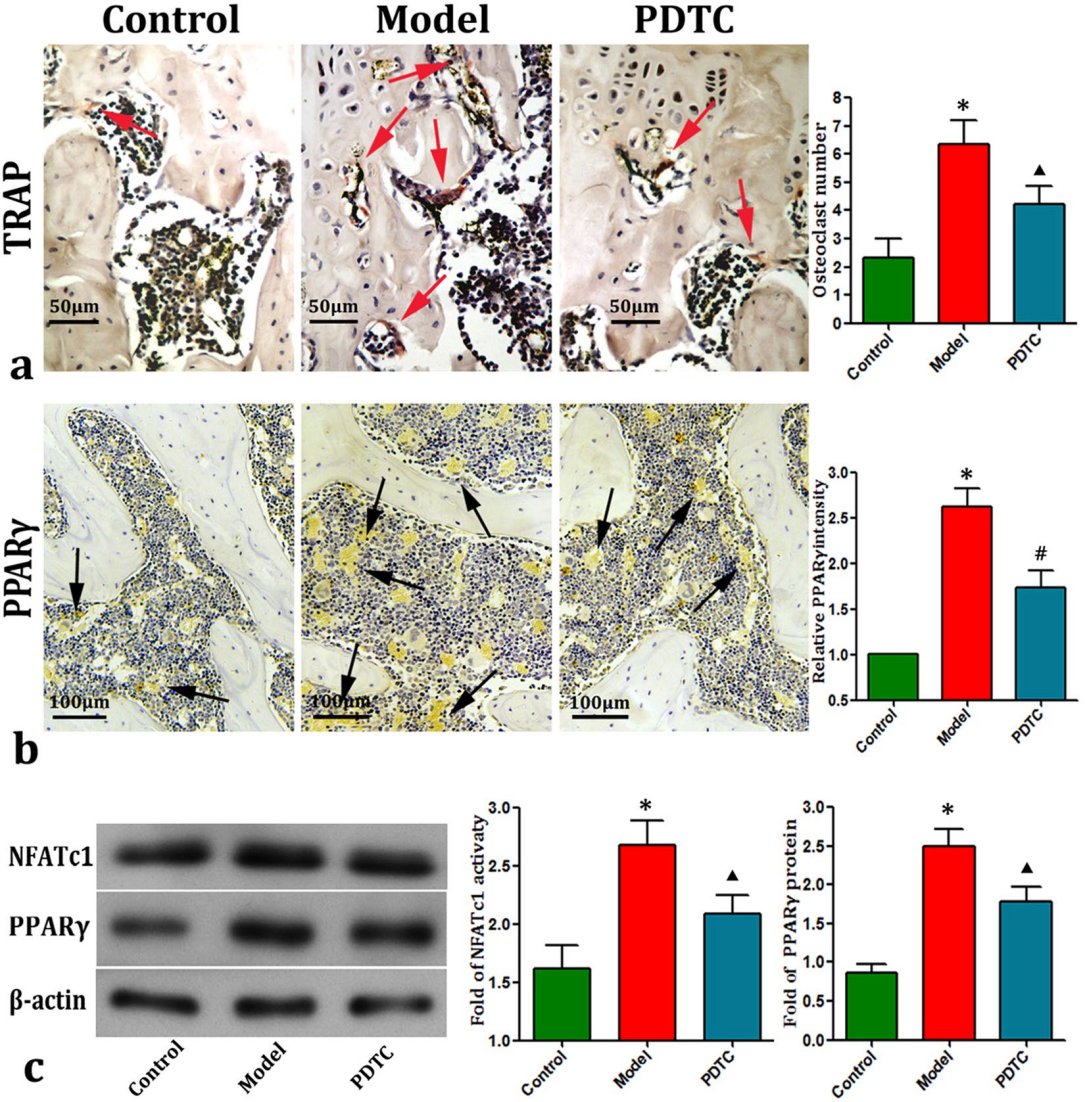
Osteoclastogenesis and adipogenesis. (**a** & **b**) TRAP and PPARγ staining. Osteonecrosis induction significantly elevated the number of osteoclast (TRAP staining, red or red brown as red arrows marked) and the number of adipocyte (PPARγ staining, yellow bubbles as blank arrows marked) in the model group, while, PDTC treatment prevented such increase effectively. Bar graphs represent the mean optical density of TRAP and PPARγ in bone marrow. (**c**) Expression levels of NFATc1 and PPARγ by Western blot. The activations of NFATc1 and PPARγ were increased after administration of LPS plus MPS. Conversely, PDTC treatment reduced the increase expressions of NFATc1 and PPARγ significantly. (Above staining quantitative analysis parameters were based on at least 10 fields <n=10**>**. Data from RT-qPCR illustrated are from three separate experiments. Bars show relative values of three groups. *p < 0.01 v.s. the control; ^▲^P < 0.05 vs. model & control; ^**#**^p < 0.05 vs. model, P < 0.01 vs. control. control. Further tested by Dunnett-t).

TRAP staining and immunohistochemistry staining of PPARγ (Fig. 5) demonstrated that ostenecrosis induction significantly increased osteoclastogenesis and adipogenesis. Consistently, Western blot analysis results showed that LPS with methylprednisolone (MPS) increased the local expression of NFATc1 and PPARγ in the femoral heads. Nevertheless, PDTC treatment effectively prevented the enhancement of bone resorption (all P < 0.05).

### PDTC inhibited osteocyte apoptosis

**Figure 6:**
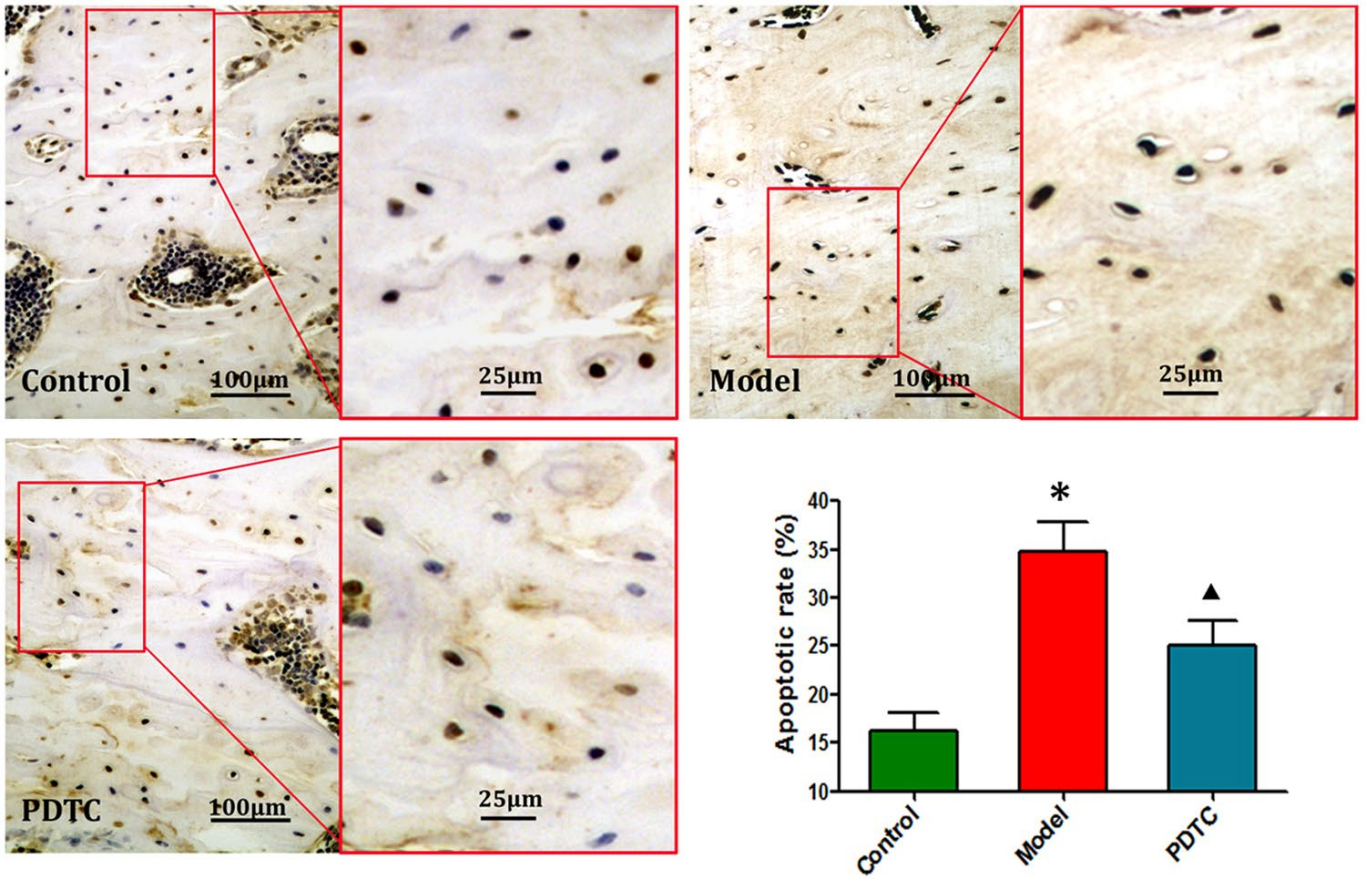
TUNEL apoptosis detection. In the model group, extensive TUNEL-positive cells, including osteocytes, chondrocytes and bonemarrow cells, were observed in the rats’ femoral heads. However, relatively lower numbers of apoptotic cells were observed in the PDTC group. Bar graphs represent the comparison of apoptotic rate. (Semi-quantitative was based on at least 10 fields per rat. *p < 0.01 v.s. the control; ^▲^P < 0.05 vs. model & control. n = 10. Tested by Dunnett-t.).

As shown in Fig. 6, many apoptosis-positive osteocytes were observed. The mean apoptotic rate in the model group was 34.25 ± 4.53%. A relatively lower number of TUNEL-positive osteocytes and a lower mean apoptotic rate (24.53 ± 3.17%) were observed in the PDTC group compared with the model group.

## Discussion

Corticosteroid treatment is a common risky factor associated with osteonecrosis^31^. High dose and prolonged corticosteroid utilization for the therapeutic of autoimmune diseases and other inflammatory diseases often lead to a series of physiological and pathological changes, such as hyperlipidemic state, abnormal coagulopathy, microvascular occlusion, bone marrow fat-cell packing, and subsequently high intraosseous pressure^32,33^. These changes all result in a high risk for necrosis; if not treated properly, they could destroy the patient’s hip joint’ activities gradually^34^. Nevertheless, confining steroid utilization to prevent such diseases has not been established yet^35^. Hence, exploring and developing promising targeted therapeutic schemes to elucidate the pathogenesis is crucial.

LPS, as a critical component of the outer membrane of bacteria, stimulates TLR4/NF-κB, which determines inflammation and a series of cascades of pathological lesions ongoing or ceased^36^, and induces acute injuries through the overproduction of numerous pro-and anti-inflammatory cytokines^37^, Thus, NF-κB inhibition ablates severity injuries and the extent of immune response through downregulating the overexpression of cytokines (IL-6 and TNF-α). Also, this inhibition also prevents bone destruction by increasing bone-forming osteoblasts and decreasing bone-resorbing osteoclasts and apoptoticrate^38^. Further studies accurately indicated that, in bone biology, osteoblastic ROS17/2.8 cells, when transfected with NF-κB, could remarkably decrease 1,25(OH)_2_ D_3_-stimulated transcription^11^. Additionally, studies on rodent models revealed that targeting block NF-κB exhibits dual benefits both in diminishing inflammation and enhancing bone regeneration^36^. In the current study, the TLR4/NF-κB pathway and inflammation (IL-6, IL-10, and TNF-α) significantly increased after osteonecrosis induction by LPS with MPS. Osteoclastogenesis, adipogenesis (TRAP and PPARγ), and osteocyte apoptosis (TUNEL) also evidently elevated in the model group. However, the inflammation response (IL-6, IL-10, and TNF-α) and these elevated bone resorption abilities were all effectively prevented or decreased when PDTC blocked NF-κB activation at the onset.

Wnt pathways control cell fate through regulating their proliferation, apoptosis, differentiation, and metabolism, which are responsible for homeostasis and different diseases^14^. The canonical Wnt/β-catenin pathway plays a key role in bone biology and regulates bone development and remodeling, whose aberration results in disturbances in bone mass^18^. For instance, Wnt-5A plays an essential role in IL-1β-mediated cartilage destruction^39^. Other evidence suggested that the genesis of SANFH is closely connected with its degradation and that of its secondary cascade target genes related to osteogenesis, angiogenesis, and adipocyte^40–42^. In the present experiment *in vivo*, PDTC administration blocked NF-κB activation, relieved inflammatory response and necrosis incidence, and upregulated the Wnt pathway and subsequent osteogenesis and angiogenesis concurrently. Tarapore^43^ similarly showed that inflammatory signals stimulate NF-κB to inhibit β-catenin and Runx2 binding to nearby consensus sites and reduce the expression of matrix proteins. Liu^44^ further demonstrated in a mouse orthopedic study that a singleton therapeutic agent for osteoporosis can gain a coupled effect on the stimulation of bone formation by the Wnt/GSK3β/β-catenin pathway and on the suppression of bone resorption by the NF-κB/c-fos/NFATc1 pathway. The most recent result of Zhao^45^ directly indicated that inhibiting NF-κB down regulates inflammatory cytokine release, enhances osteoblast differentiation, and stimulates the Wnt3a/β-catenin pathway; these results agree with the present one.

Combining these results, we conclude that the excessive activation of TLR4/NF-κB may interactively suppress the Wnt/β-catenin pathway in an early stage; subsequently affect their cascade target genes as osteogenesis, angiogenesis, and adipocyte differentiation; and contribute to the genesis and development of SANFH. Such crosstalk effect exists in different forms; during prolonged inflammation, cytokines are closely associated with increased apoptosis; in addition, Wnt inhibition is closely linked to facilitate apoptotic effects^46,47^. Thus, both up-and-down regulation of TLR4 and Wnt pathways cooperate and exacerbate the dysregulation of the apoptotic pathway, thereby contributing to the loss of bone mass. Furthermore, the Wnt pathway exerts a positive effect on anti-inflammatory activity^26^. Although cytokines may not directly correspond to the pathogenesis of SANFH, the incapacitation of anti-inflammatory activity may exacerbate the hemodynamic changes and result in redouble blood hypercoagulability^48^; in addition to the disorders of bone homeostasis between formation and destruction, the disequilibrium may be aggravated, and the tendency to necrosis may increase eventually.

DKK1, as a powerful antagonist of the canonical Wnt signaling pathway, plays a key role in the remodeling of joints, and its serum concentration can indirectly reflect the activation of Wnt pathways. DKK1 is also significantly high in patients with osteoporosis^49,50^. Consistently, in the present study, the serum secretion of DKK1 considerably increased after osteonecrosis induction and effectively decreased after PDTC treatment. Swarnkar^12^ similarly detected that NF-κB activation inhibits osteogenic markers and stimulates anti-osteogenic factors (DKK1). Fulciniti^51^ found that, in reverse crosslinking, anti-DKK1 (BHQ880) increases β-catenin expression, reduces NF-κB and IL-6 secretion, and ultimately increases osteoblast and trabecular in SCID–hu murine. Thus, in addition to GSK-3β^52,53^, the expression levels of DKK1 in the present study further indicated other factors that correlate with TLR4/NF-κB and Wnt/β-catenin activation and probably link to the genesis of SANFH. Combining previous evidence^25,26,54,55^ and the present result, we proposed the effects of schematic model net between the TLR4/NF-κB and Wnt/β-catenin pathways on the explanation of the genesis and development of SANFH in SD rats *in vivo* (Supplementary materials Fig. 0).

The limitations of the present study is that, although we explored and evaluated the interactive effects of the two pathways on several indicators, other factors, such as lipid (TG, TC, and HDLC) metabolism, were not monitored. Moreover, the cost and inflammatory factors were only explored only at three time points. Further work should be conducted before such crosstalk effect-targeted therapeutic treatments were unequivocally and skillfully utilized in clinical practice. Definite experiments, such as knock-in and -out of relevant genes, should also be performed for further evidence. In future research, different pharmaceuticals targeting different interactive pathways or points at different pathological periods may be dynamically utilized alone or in combination and comprehensively evaluated to explore the optimal therapeutic period for preventing the genesis and development of SANFH.

## Conclusion

Our findings suggest that PDTC targets block NF-κB effectively and ameliorate osteonecrotic changes. These targets play important role in transforming bone decompensation to bone regeneration, and improve the microstructure of the femoral head ultimately, which should be closely connected with the rectification of the imbalanced crosstalk effect between the TLR4/NF-κB and Wnt/β-catenin pathways and the further disturbance of their cascade target genes in osteogenesis, angiogenesis, osteoclastogenesis, adipogenesis, and apoptosis. PDTC treatment.

## Materials and Methods

### Reagents and animals

LPS, MPS, and PDTC were purchased from Sigma–Aldrich (St. Louis, MO, USA). Antibodies of TLR4, total NF-κB-p65, anti-OPG, anti-Runx2, PPARγ, and anti glyceraldehyde-3-phosphate dehydrogenase (GAPDH) were obtained from Cell Signaling Technology, Inc.(Danvers, MA, USA). The enzyme-linked immunosorbent assay (ELISA) kits for the determination of IL-6, IL-10, and TNF-α were produced by Nanjing KeyGEN Biotech. Co., Ltd. (Nanjing, China). NFATc1,Wnt3a, and β-catenin antibody and isotype IgG control antibody were obtained from Santa Cruz Biotechnology(Santa Cruz, CA, USA).

Eighty adult male Sprague–Dawley (SD) rats (aged 10–12 weeks, weighing 270–330 g) were purchased from the Experimental Animals Center of Xi’an Jiaotong University. All rats were housed in a light controlled environment (12 h light–dark cycles) under standard temperature (22 ± 3 °C) and a relatively suitable humidity (50% ± 15%). The environment was also equipped with an air-filtering system. The cages and water were sterilized, and the animals were allowed activity freely. Standard rodent chow and water ad libitum were provided. All animal experiment procedures were approved under the guidelines of the Laboratory Animal Research Committee and conducted in accordance with the accepted policies of our university and Chinese animal care authorities, in addition to the National Institute of Health Guide for the Care and Use of Laboratory Animals.

### Experimental groups and protocols

After a week of feeding adaptation, the animals were accurately weighed and randomly divided into three groups by using the random number table method (20 rats for the control group, and 30 rats for the model and PDTC groups). During the whole process, the serum concentrations of cytokines and DKK1 were dynamically monitored to reflect the TLR4/NF-κB and Wnt/β-catenin pathways directly or indirectly. Six blood samples from each group were obtained from randomly selected rat tail vein at 1, 2, and 6 weeks. Prior to the sacrifice, three rats from each group were randomly selected for intra-arterial ink perfusion. The SANFH model was established as previously described by Okazaki^6^. All rats were treated as follows:

### Model group (LPS + MPS plus saline)

LPS was injected at 2 mg/kg/day via tail intravenously for two times. Subsequently, MPS (40 mg/kg) was daily injected into the left gluteus muscle of the rats for three times on days 3, 4, and 5. The isovolumetric of saline was injected intraperitoneally (i.p.) compared with the element in the PDTC group.

### Prevention group (LPS + MPS plusPDTC)

In addition to the injection of LPS and MPS, the prevention group received 14 doses of PDTC (from day 1 to day 14, 100 mg/kg, Sigma Chemical Co. St. Louis, MO, USA) via daily i.p. injection concurrently or alone. The PDTC dosage for each animal was adjusted according to the fluctuant body weight every 3 days.

### Sham group (saline + saline plus saline)

Rats in the sham group underwent a similar administration of equal volume saline per time.

### Tissue sample preparation

Rats from each group were sacrificed 4 weeks after the last PDTC injection (six weeks totally). All animals were decapitated after a thorough anesthesia by pentobarbitone (100 mg/kg). The femoral heads were obtained and dissected, and soft tissue of excised femurs were removed. Afterward, the left ones were fixed in 4% paraformaldehyde solution (pH 7.4) for 3 days. After decalcification, the tissues were embedded in paraffin, cut into 5μm-thick slices along the coronal plane with a microtome, and then stained with hematoxylin and eosin (HE). The right sides were stored at −80 °C for Western blot analysis and reverse transcription quantitative real-time polymerase chain reaction (RT-qPCR) test.

### Histopathological staining and evaluation of SANFH

Routine HE and Masson staining were performed to assess the general architecture and injury of the tissue. Sections of slices were viewed at different magnifications and photographed with a microscope (Olympus, Tokyo, Japan). Images were analyzed by Image-Pro-plus 6.0 software (Media Cybernetics, Baltimore, MD). The osteonecrotic changes and repair processes in steroid-treated rats were observed by histopathological examination using a light microscope. The evaluation criteria for osteonecrosis were based on a previous report^30^. Three experienced observers evaluated all slides in a blinded fashion. Osteonecrosis was evaluated when microfracture necrosis, accumulation of hypertrophy fat cells, debris of bone marrow cells, and condensed nuclei in osteocytes were observed.

### Immunohistochemistry and TRAP staining

The remaining femoral head tissue slices obtained were processed immunohistochemically to detect β-catenin, OPG, CD31, PPARγ, and TRAP staining to osteoclasts. Positive staining for the signaling molecules in the femoral heads would be visible as (reddish) brown puncta and bundles distributed in the bone marrow, periosteum, and bony trabeculae. Samples without primary antibodies were used as negative controls. The intensities of immunostaining in the three groups were quantitatively analyzed.

### Terminal deoxynucleotidyl transferase-mediated dUTP nick end labeling (TUNEL) apoptosis detection

TUNEL assays were used to observe the presence and location of apoptosis. The TUNEL detection kit (Promega Co. Ltd., Beijing, China) was used in accordance with the manufacturer’s instructions. Cells whose nuclei were brown or brown-yellow or whose cytoplasm included a few brown or brown-yellow granules were interpreted as positive. The sections were observed and calculated in each section by two researchers blindly.

### Intra-artery ink perfusion

To measure the blood supply of the femoral head, selective vascular perfusion with Indian ink was performed 6 weeks after the induction of osteonecrosis. Finally, three animals from each group were perfused with 20 mL of India ink as previously desribed^56^. Afterward, all animals were sacrificed 24 h after refrigeration under 4 °C, and thebilateral thighbones were harvested. All samples were fixed, decalcified, embedded, and then cut into 5μm-thick slices. The perfusion ratio of slices was calculated by using the Image-Pro Plus 6.0 image analysis software. The perfusion ratio was defined as the ratio of the area of the inked artery to the area of the entire femoral head^57^.

### Hematological examination

To detect the dynamic changes in the TLR4/NF-κB and Wnt/β-catenin pathways directly or indirectly, the serum levels of cytokines (IL-6, IL-10, and TNF-α) and DKK1were detected with a commercially available multiplex suspension ELISA (Biomart, Wuhan, China) at 1, 2, and 6 weeks. All experimental processes were performed in accordance with the manufacturer’s protocols. Unknown concentrations were calculated by a standard curve derived from recombinant cytokine standard, and all samples were run in duplicate. Final results were read within 15 min spectrophotometrically at λ = 450 nm. All values were subtracted by the mean value of the zero standard before result interpretation. Finally, values were expressed as pg/mL protein, and the standard curve was plotted according to the amount in all samples using GraphPad Prism 5.0 software.

### Calcein and tetracycline double-labeling

Calcein (CAL) and tetracycline (TCY) double labels (10-day interval) were administered with CAL (12 mg/kg, subcutaneously) at day 28 and TCY (250 mg/kg, p.o.) at day 38. Finally, the femurs were collected. Comparison among groups was made through histomorphometry as previously reported^58^.

### RT-qPCR analysis

The RNA isolation and RT-qPCR assay were carried out in accordance with the manufacturer’s protocol. In brief, total RNA was extracted with TRIzol® reagent (Invitrogen, Carlsbad, CA, USA) from the tissue homogenates. The total RNA (3 μg) was reverse transcribed to cDNA using the QuantiTect Reverse Transcription Kit (Thermo Fisher Scientific Inc., Fremont, CA,USA). After further procedure, the gene expression levels of TLR4, NF-κB p65, Runx2, and VEGF were normalized to GAPDH mRNA level. The relative mRNA was calculated using the comparative 2^**−**ΔΔCt^ method. PCR primer pairs were selected from different exons of the corresponding genes, as shown in Table 1.

**Table 1:** PCR primer pairs sequence of SD rat.

Target gene	Pairs	Primer pairs sequence (5′-3′)
rat TLR4	forward	5′-ggcatcatcttcattgtccttg-3′
	reverse	5′-agcattgtcctcccactcg-3′
rat NF-κB p65	forward	5′-tgcaggctcctgtgcgagtg-3′
	reverse	5′-tccggtggcgatcgtctgtgt-3′
rat Runx2	forward	5′-gttatgaaaaaccaagtagccaggt-3′
	reverse	5′-gtaatctgactctgtccttgtggat-3′
rat VEGF	forward	5′-ttgtttctgggattcctgtag-3′
	reverse	5′-ccaactcaagtccacagcag-3′
rat GAPDH	forward	5′-atggtgaaggtcggtgtgaacg-3′
	reverse	5′-cgctcctggaagatggtgatgg-3′

### Protein extraction and Western blot analysis

Total proteins of the femoral head tissue were extracted, and equal amounts of proteins (75 μg) were separated using sodium dodecyl sulfate-polyacrylamide gel electrophoresis and blotted onto polyvinylidene difluoride membranes. All antibodies for Western blot analysis were diluted at a concentration of 1:200. Blots were incubated for 24 h with antibodies against TLR4, NF-κB p65, Wnt3a, β-catenin, and c-Myc diluted in Tris-buffered saline with 0.1% Tween-20 plus 5% (w/v) fat-free milk. Proteins were normalized to GAPDH or β-actin and expressed as % change in protein level compared with CON-0 set as a ratio.

### Micro-computed tomography (μ-CT) imaging and selection of the region of interests (ROIs)

The μ-CT scan was performed to validate the necrotic change in different groups. Prior toμ-CT scanning, the femurs were thawed to room temperature (25 °C). The right distal femoral metaphysis was scanned using the Micron X-ray 3D Imaging System (Y.Cheetah, YXLON International GmbH, Germany). The scan resolution was set at 10 µm, and an average pixel size of 7 µm was used for scanning. These images were reconstructed in three dimensions for the presentation of SANFH necrosis and structural integrity of the trabecular bone. The ROIs were located 0.3 mm away from the cartilage notch between the calotte and the neck, which covered a cylinder (Fig. 1A; r = 0.65 mm; 0.3mm thick). After selection, extraction, and coloring, the ROIs were smoothened twice at a replaced condition of strength 3. Finally, the bone volume faction (BV/TV), trabecular number (Tb.N), trabecular separation (Tb.Sp), and trabecular thickness (Tb.Th)were obtained for microstructure analysis.

### Statistical analysis

One-way ANOVA and further Dunnett’s test were performed to determine significant differences in histopathological staining, mRNA expression, and protein level among different groups. The chi-square test was carried outto determine significant differences between osteonecrosis and nonosteonecrosis. SPSS version 18.0 for Windows (SPSS Inc., Chicago, IL, USA) was used. All data are presented as the mean ± SD. Results were considered statistically significant at P < 0.05.

## Electronic supplementary material

Supplementary materials
