# Supplementary material for: Excessive Activation of TLR4/NF-κB Interactively Suppresses the Canonical Wnt/β-catenin Pathway and Induces SANFH in SD Rats
Source: Sci Rep. 2017 Sep 20;7:11928. doi: 10.1038/s41598-017-12196-8 (PMC5607349; doi:10.1038/s41598-017-12196-8)

Excessive Activation of TLR4/NF-κB Interactively Suppresses the Canonical Wnt/β-catenin Pathway and Induces SANFH in SD Rats*

Junpeng Pei[1], Lihong Fan[1#], Kai Nan[1], Jia Li[2], Zhibin Shi[1], Xiaoqian Dang[1], Kunzheng Wang[1]

[1] **Department of Orthopaedics, the Second Affiliated Hospital of Xi'an Jiaotong University, No. 157 Xiwu Road, Xi'an710004, Shaanxi Province, People's Republic of China**

[2] **Department of Orthopaedics, First Affiliated Hospital of Xi'an Jiaotong University, School of Medicine, Xian 710061, China**

*This study was supported by the National Natural Science Foundation of China (No. 81371944 and 81572145) and the Fundamental Research Funds for the Central Universities. All authors read and approved the final manuscript. The authors have declared they have no conflicts of interest in the study.

Jun-peng Pei, E-mail: pei_junpeng @163.com

#Corresponding author: Li-hong Fan,

E-mail: drfan2140@163.com / drfan2140@mail.xjtu.edu.cn

Junpeng Pei: pei_junpeng @163.com

Lihong Fan: drfan2140@163.com / drfan2140@mail.xjtu.edu.cn

Kai Nan: 1010641217@qq.com

Jia Li: happylee_xjtu@163.com

Zhibin Shi：jackky9999@sohu.com

Xiaoqian Dang: dangxiaoqian@sohu.com

Kunzheng Wang: wkzhl1955@mail.xjtu.edu.cn

Fig.0

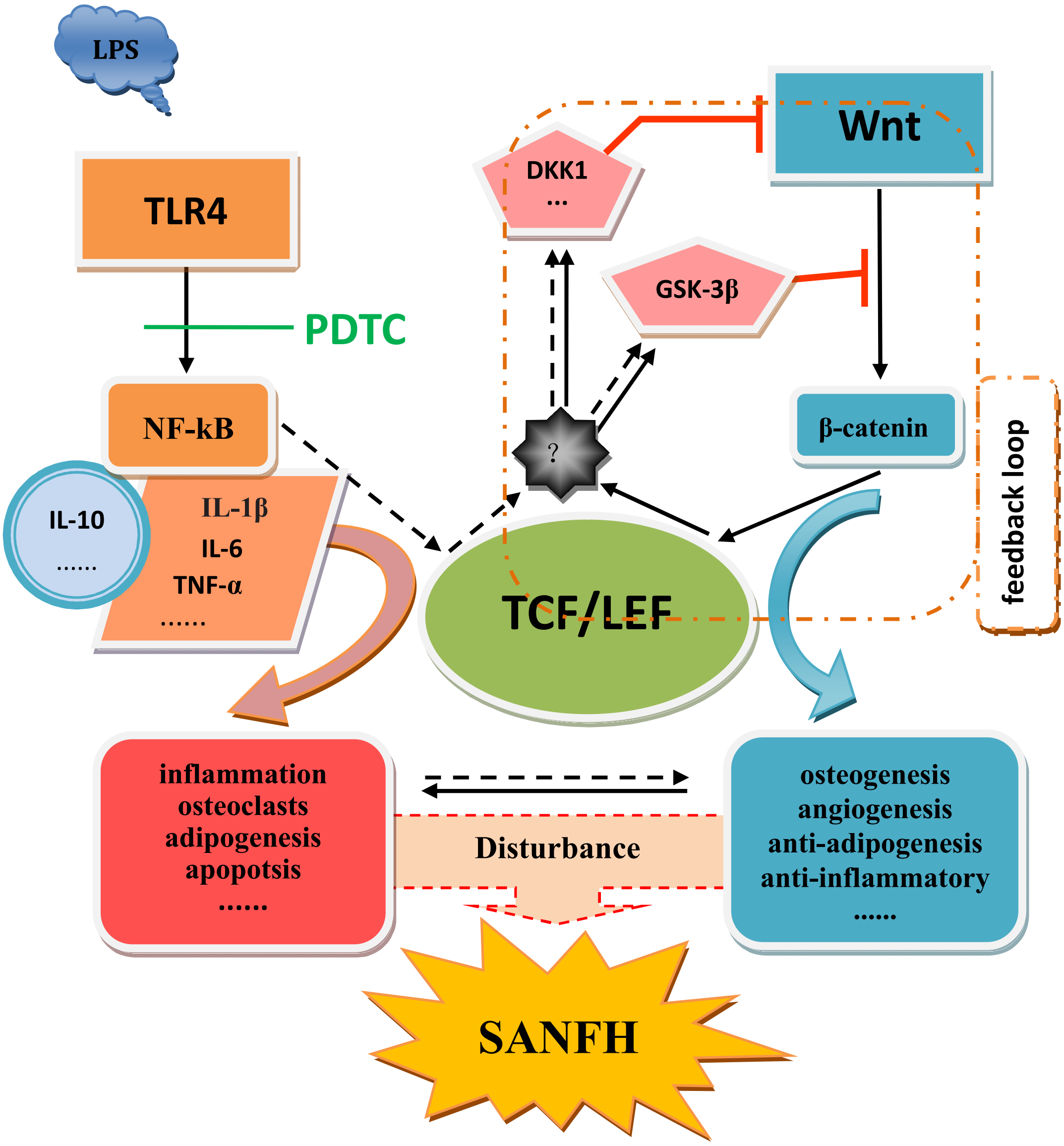

Fig.0 Proposed schematic model. In this model, NF-κB is sustained activated after induction of SANFH, which may interactively elevates the expression of DKK1 and subsequently inhibits Wnt/β-catenin pathway. Thus, excessive NF-κB combine to the suppressed Wnt/β-catenin inhibits osteogenesis and angiogenesis, promotes osteoclastogenesis and adipogenesis as well as apoptosis further, and eventually leads to osteonecrosis.

Orignal Western blot images

Fig.2.B1

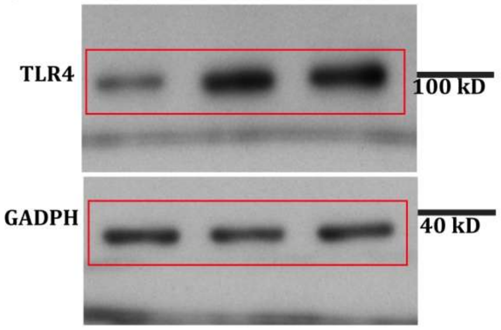

Fig.2.B2

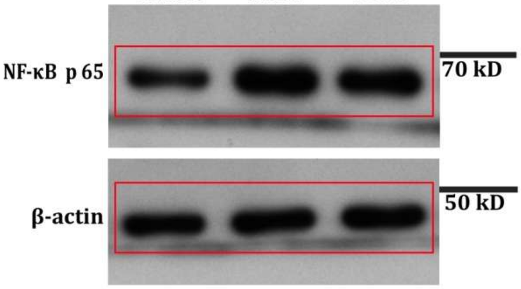

Fig.3.b

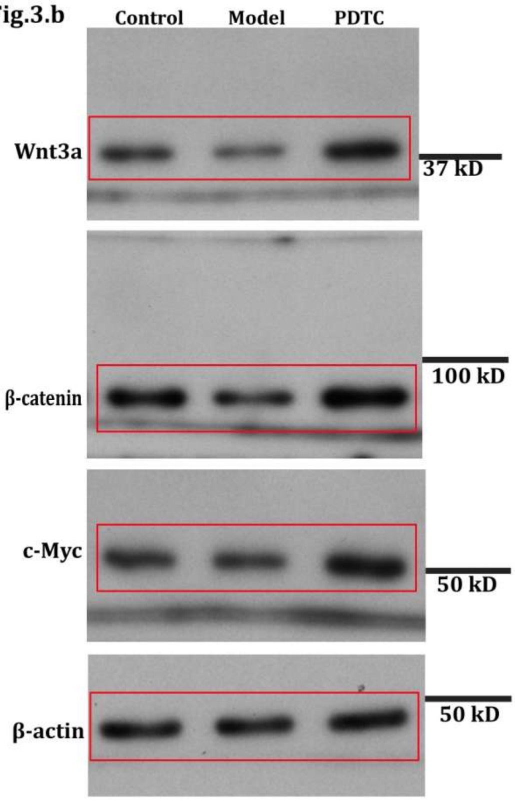

Fig.5.c

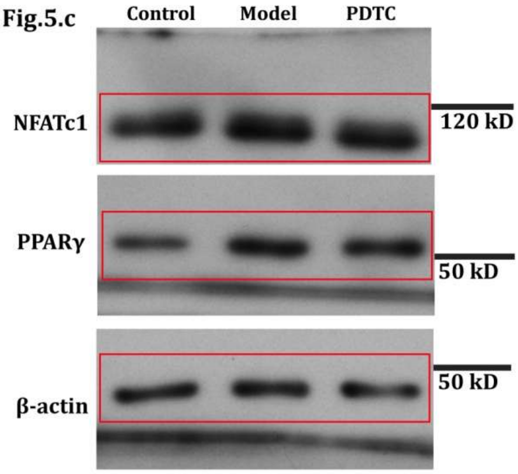

Supplement: Supplementary file 1 — Supplementary materials [file 41598_2017_12196_MOESM1_ESM.pdf]
